# Effects of Symptom Burden on Quality of Life in Patients with Lung Cancer

**DOI:** 10.3390/curroncol31100458

**Published:** 2024-10-12

**Authors:** Ling-Jan Chiou, Yun-Yen Lin, Hui-Chu Lang

**Affiliations:** 1 Department of Healthcare Administration, Asia University, Taichung 413305, Taiwan; lingjan@asia.edu.tw; 2 Department of Performance, Taipei Medical University-Shuang Ho Hospital, Ministry of Health and Welfare, Taipei 115204, Taiwan; ellainelin33212@gmail.com; 3 Institute of Hospital and Health Care Administration, National Yang Ming Chiao Tung University, Taipei 112304, Taiwan

**Keywords:** lung cancer, quality of life, symptom burden, EORTC QLQ-C30, EORTC QLQ-LC13

## Abstract

Lung cancer patients suffer from numerous symptoms that impact their quality of life. This study aims to identify the symptom burden on quality of life in lung cancer patients. This survey used a structured questionnaire to collect data from 8 March 2021 to 12 May 2021. Patient demographic information was collected. The data on symptom burden and quality of life (QOL) of patients were obtained from the QLQ-C30 and the QLQ-LC13. The stepwise multiple regression analysis was used to estimate lung cancer-related symptom burden in relation to quality of life. The study included 159 patients with lung cancer who completed the questionnaire. The mean age of the patients was 63.12 ± 11.4 years, and 64.8% of them were female. The Global Quality of Life score of the QLQ-C30 was 67.87 ± 22.24, and the top five lung cancer-related symptoms were insomnia, dyspnea, and fatigue from the QLQ-C30, and coughing and dyspnea from the QLQ-LC13. The multiple regression analysis showed that appetite loss was the most frequently associated factor for global QOL (β = −0.32; adjusted R^2^: 27%) and cognitive function (β = −0.15; adjusted R^2^: 11%), while fatigue was associated with role function (β = −0.35; adjusted R^2^: 43%), emotional function (β = −0.26; adjusted R^2^: 9%), and social function (β = −0.26; adjusted R^2^: 27%). Dyspnea was associated with physical function (β = −0.45; adjusted R^2^: 42%). Appetite loss, fatigue, and dyspnea were the main reasons causing symptom burdens on quality of life for lung cancer patients. Decreasing these symptoms can improve the quality of life and survival for patients with lung cancer.

## 1. Introduction

Lung cancer is the most common cancer and the leading cause of death in the world [1,2]. Patients with lung cancer experience multiple symptoms that are highly disruptive to their physical and emotional functioning and quality of life [3,4]. These symptom clusters are caused by lung cancer itself and the side effects of treatments such as chemotherapy or radiotherapy, and interfere with patients’ daily life functions and affect their quality of life [5].

Confirming the phenomenon of symptom clusters among lung cancer patients may provide physicians with the ability to identify disease conditions during the treatment process [6,7] and provide a reasonable treatment goal to reduce the effect of symptoms while increasing the functioning and quality of life (QOL) for patients [8]. Furthermore, improving lung cancer-related symptoms and quality of life may affect survival [9,10,11]. The literature has found that fatigue, loss of appetite, cough, pain, and shortness of breath were significant predictors of the quality of life in patients with lung cancer [12,13,14,15,16].

To identify the symptom burden during the treatment process in patients with lung cancer, healthcare providers should encourage using multidimensional strategies to manage the main symptoms and improve quality of life [15,16]. Previous studies have only focused on the significant symptom predictors of the quality of life. Some studies found that overall symptom severity was the main negative predictor of quality of life [17,18]. Few studies have examined the explained amount of variance in single symptoms for quality of life among lung cancer patients to identify the main symptom that affected QOL.

Given the significance of treatment strategies and the limited outcomes observed in prior research, we pursued research to define and assess the main burden of lung cancer-related symptoms and quality of life in lung cancer patients. These findings may help identify the symptom burden conditions for physicians to develop effective healthcare interventions to improve healthcare quality and enhance the quality of life and survival of lung cancer patients.

## 2. Methods

### 2.1. Design and Sample

Patients were recruited at a thoracic oncology department at a medical center. The inclusion criteria were patients diagnosed with lung cancer, aged above 20 years, and fully conscious. Patients were introduced to the interviewer by a physician. After the study was explained and informed consent was obtained, a one-on-one, face-to-face survey was conducted from 8 March 2021 to 12 May 2021. A total of 159 completed questionnaires were collected.

### 2.2. Patient Demographics and Database

The questionnaire covered the following sections: patient demographic information (i.e., gender, age, marital status, education level, living with family, diagnosis year, smoking status, family history, secondhand smoking status, disease stage, income level, occupational status, and treatment history, including nil, chemotherapy [CT] or radiotherapy [RT], or CT and RT). The data on symptom burden and quality of life (QOL) were obtained from the QLQ-C30 and the QLQ-LC13, which have good validity and reliability [19].

### 2.3. EORTC Quality of Life Questionnaire C-30 (EORTC-QLQ-C30)

The EORTC QLQ-C30, which comprises 30 items, is the most popular instrument applied to measure quality of life for all kinds of cancers [20]. It contains six primary QOL domains: global health status scale (2 items), physical function (5 items), role function (2 items), emotional function (2 items), cognitive function (2 items), and social function (2 items); eight cancer symptoms (i.e., fatigue, nausea and vomiting, pain, dyspnea, insomnia, appetite loss, constipation, and diarrhea); and a financial difficulties scale. The subjects responded to each statement on a scale of 0 (‘not at all’) to 4 (‘very much’). The global health status scale ranged from 0 (‘very poor’) to 7 (‘excellent’). The scores were calculated by first computing the raw scores as the means of item responses. The raw scores were then converted into scaled scores ranging from 0 to 100. Higher scores for the scales indicate a better QOL. A higher average score equates to a greater symptom burden [21].

### 2.4. EORTC Quality of Life Questionnaire in Lung Cancer (EORTC-QLQ-LC13)

The 13 items that the QLQ-LC13 questionnaire measures include 10 lung cancer-related symptoms and treatment-related adverse effects (i.e., dyspnea, coughing, hemoptysis, sore mouth, dysphagia, peripheral neuropathy, alopecia, chest pain, pain in the arm or shoulder, pain in other parts). The QLQ-LC13 items use a 1-to-4 verbal response scale. The scores were calculated by first finding the means of item responses to obtain the raw scores, which were then converted into scaled scores ranging from 0 to 100. Higher scores for the scales indicate a greater burden for symptom scales [22].

### 2.5. Statistical Analysis

Descriptive statistics were presented for sample characteristics. The frequency and percentage or mean and standard deviation were computed for categorical and continuous variables. Correlation analyses were performed to examine the associations between lung cancer-related symptoms and quality of life variables. The stepwise multiple regression analysis was conducted to identify the factors influencing the six primary QOL domains. The variance inflation factor (VIF) was used as an indicator for multicollinearity effects. In our study, the level of VIF of all variables was less than 4, which was the acceptable level [3,7,23]. The standardized betas (β) and explained variation (Adjusted R^2^ and R^2^ change) of significant variables were used to estimate the effects of lung cancer-related symptoms for QOL. Analyses were performed using IBM SPSS version 25.

## 3. Results

Of the 159 patients, the mean age was 63.12 ± 11.4 years, and 64.8% of all patients were female. The majority of the patients were married (76.7%), had completed high school or higher education (61.6%), lived with family (85.5%), had been diagnosed within five years (68.6%), had never smoked (78.0%), had no family history of smoking (73.6%), had no secondhand smoke status (73.0%), had > US$645 monthly income level (59.8%), and were unemployed (79.2%). Most had been diagnosed with stage IV (68.5%) lung cancer and were receiving chemotherapy or radiotherapy (42.1%), with a proportion undergoing second-line treatment (34.6%) as their treatment (Table 1).

Table 2 shows the quality of life and lung cancer-related symptoms data collected from the QLQ-C30 and the QLQ-LC13. The Global QOL score was 67.87 ± 22.24. The mean ± SD of the five function scores was 82.14 ± 20.28 for physical function (PF), 82.29 ± 27.98 for role function (RF), 86.69 ± 21.26 for emotional function (EF), 84.28 ± 21.24 for cognitive function (CF), and 80.29 ± 27.55 for social function (SF). The top five highest scores of lung cancer-related symptoms were insomnia (32.08 ± 36.72), dyspnea (24.53 ± 31.48), and fatigue (23.27 ± 27.04) among the nine symptoms from the QLQ-C30 and coughing (23.06 ± 29.04) and dyspnea (19.92 ± 27.28) among the ten symptoms from the QLQ-LC13.

Table 3 was the result of Pearson correlations between symptoms and quality of life variables. The global QOL had negative correlations with fatigue (r = −0.49, *p* < 0.01), nausea and vomiting (r = −0.029, *p* < 0.01), pain (r = −0.36, *p* <0.01), dyspnea (r = −0.34, *p* <0.01), appetite loss (r = −0.52, *p* < 0.01), constipation (r = −0.28, *p* < 0.01), diarrhea (r = −0.25, *p* < 0.01), financial difficulties (r = −0.35, *p* < 0.01), dyspnea (r = −0.44, *p* < 0.01), alopecia (r = −0.17, *p* < 0.05), chest pain (r = −0.37, *p* < 0.01), pain in the arm or shoulder (r = −0.17, *p* < 0.05), and pain in other body parts (r = −0.20, *p* < 0.05). In the other five function scores, the significant symptom variables were shown to be negatively correlated, and the symptoms with the highest r were fatigue, pain, dyspnea, appetite loss, dyspnea, and chest pain.

**Figure 1:**
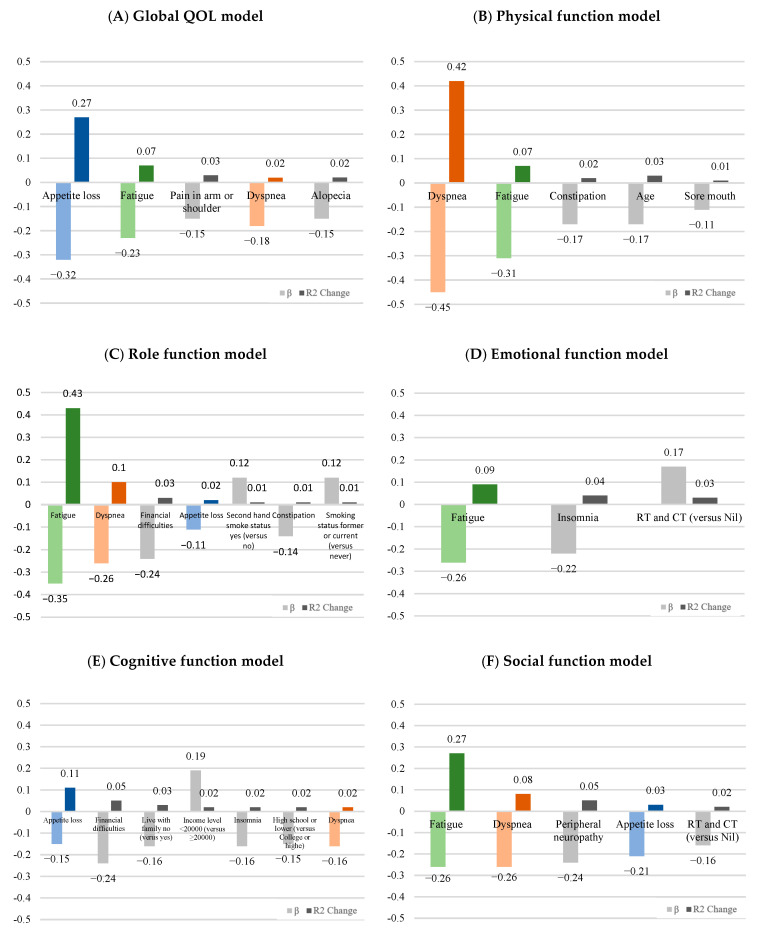
The standardized betas (β) and explained variation (R^2^ change) of significant symptoms for (**A**) global health, (**B**) physical function, (**C**) role function, (**D**) emotional function, (**E**) cognitive function, and (**F**) social function according to the result of the stepwise multiple regression analysis. Appetite loss (blue), fatigue (green), and dyspnea (orange) were the main symptom burdens in lung cancer patients.

In Table 4, the stepwise multiple regression analysis revealed that the predictors of global QOL were appetite loss, fatigue, pain in arm or shoulder, dyspnea, and alopecia, which accounted for 40% of the variance and the β from −0.15 to −0.32 (F = 22.02, *p* < 0.001). Appetite loss was the most affected factor for 27% of the variance. The variables of dyspnea, fatigue, constipation, age, and sore mouth were the predictors of physical function, which accounted for 54% of the variance and the β from −0.11 to −0.45 (F = 37.82, *p* < 0.001). The predictors of role function were fatigue, dyspnea, financial difficulties, appetite loss, and constipation, which accounted for 60% of the variance and the β from −0.12 to −0.35 (F = 35.38, *p* < 0.001). Furthermore, smoking status (β = 8.08) and secondhand smoke status (β = 7.47) were positively associated with role functioning. The significant factors of emotional function were fatigue, insomnia, and RT and CT treatment, which accounted for just 13% of the variance and the β from −0.17 to −0.26 (F = 9.03, *p* < 0.001). The variables of appetite loss, financial difficulties, life with family, income level, insomnia, education level, and dyspnea were significant with the cognitive function, which accounted for 23% of the variance and the β from −0.15 to −0.24 (F = 7.90, *p* < 0.001). The predictors of social function were fatigue, dyspnea, peripheral neuropathy, appetite loss, and RT and CT treatment, which accounted for 45% of the variance and the β from −0.16 to −0.26 (F = 26.40, *p* < 0.001). In these five function models, the symptoms of dyspnea, fatigue, fatigue, appetite loss, and fatigue had accounted for the higher variance (R2 change) individually (Figure 1).

## 4. Discussion

The findings of our study demonstrate that lung cancer patients experience a lower perception of their global health status and social functioning compared to other aspects of functioning. The burden of symptoms reported by lung cancer patients, as assessed by the QLQ-C30 and QLQ-LC13 questionnaires, primarily included insomnia, fatigue, coughing, and dyspnea. In our regression model, these lung cancer-related symptoms and factors accounted for significant variations of 40%, 54%, 60%, 13%, 23%, and 45% in global quality of life, physical functioning, role functioning, emotional functioning, cognitive functioning, and social functioning, respectively. Of particular importance among these predicted factors, appetite loss, dyspnea, and fatigue were found to exert the strongest impact on the quality of life experienced by lung cancer patients. Accordingly, prioritizing these symptoms is essential to enhance overall wellbeing and quality of life for individuals with lung cancer. These findings highlight the need for targeted interventions and personalized care strategies that address the specific challenges posed by these symptoms, ultimately leading to improved outcomes and enhanced quality of life for lung cancer patients.

In our study, the global quality of life (QOL) score was the lowest (mean ± SD: 67.87 ± 22.24) compared to the six domains of the QLQ-C30 questionnaire. However, compared to previous studies [19,24,25], our study was slightly higher than the others. These variations may be attributed to differences in disease stage among the sample populations, as well as improvements or deteriorations in functioning and QOL depending on the treatment type and follow-up duration. This highlights the need for greater emphasis on enhancing the quality of life for patients undergoing treatment. Therefore, it is worth exploring the symptoms that significantly impact the QOL of lung cancer patients.

Consistent with prior literature [4,12,15,24,26,27,28], our study observed higher scores for lung cancer-related symptoms, including insomnia, dyspnea, fatigue, and coughing, as measured by the QLQ-C30 and QLQ-LC13 questionnaires (Table 2). These symptoms were found to have a moderate to significantly low impact on various aspects of quality of life (i.e., global GOL, PF, RF, EF, CF, and SF, Table 3). Our study further found a higher explained variation of lung cancer-related symptoms in the regression model (Table 4). According to the adjusted R square, the highest-burden symptoms in patients with lung cancer were appetite loss for global QOL (27%) and cognitive function (11%); fatigue for role function (43%), emotional function (9%) and social function (27%); and dyspnea (42%) for physical function. These results provided a very important reference value for physicians in their treatment strategy of lung cancer patients. Appetite loss, dyspnea, and fatigue were the main burden symptoms. If these three symptoms can be improved, it will benefit patients’ quality of life and even affect their future survival rates. Various intervention programs, such as nutritional counseling, nutritional supplements, breathing exercises, and physical activity programs have been shown to positively influence these symptoms and quality of life in the treatment of lung cancer patients [28,29,30,31]. As previous studies have pointed out, patients with lung cancer suffer multiple symptoms caused by disease and treatment. The treatment-related symptoms increased over time, but disease-related symptoms tended to be palliated after treatment initiation [24]. When treating lung cancer patients, physicians can consider the symptom burden in treatment time to improve the patient’s QOL.

The patients undergoing active radiotherapy and chemotherapy for treatment (compared with no treatment) were better in emotional function (t = 2.23, *p* = 0.022) and worse in social function (t = −2.61, *p* = 0.010). This finding was similar to that of previous studies [24], and this may be because the patients felt reassured from receiving treatment and, thus, felt good for emotional function, but because of the side effects of the treatment, their social functioning was affected.

Previous studies have indicated the significant impact of lung cancer on patients’ health-related quality of life. Specifically, stage IV disease and line of treatment were found to substantially deteriorate utility [32]. Our analysis utilized a mixed sample approach, which underscores the complexity of these relationships. Future research should focus on examining how different subgroups of treatment lines affect symptoms and QOL. Investigating these relationships in greater detail could provide valuable insights into how various stages of treatment influence patient wellbeing and symptom management, potentially leading to more tailored and effective interventions.

A notable strength of our study is our focus on identifying the primary burden symptoms in lung cancer patients, which provides valuable insights into their specific challenges and experiences. We found that appetite loss affected global QOL and cognitive function, while fatigue impacted role, emotional, and social function. Dyspnea was a major burden for physical function, as indicated by the R square in our regression model. These findings provide healthcare professionals with critical, actionable insights that can guide targeted interventions to improve patient outcomes. Unlike previous studies that have primarily reported associations between symptoms and lung cancer, our research offers a more nuanced understanding of how these symptoms affect various dimensions of patient wellbeing.

Despite the significant findings in this study, there are some limitations in our study. Firstly, the data for our study were derived from a medical center in Taiwan, and the results cannot be generalized to all patients with lung cancer. Additionally, the population is predominantly female, nonsmoking, and with high education and income levels, which may affect the applicability of our findings to other demographic groups [33]. Secondly, the samples were from patients who agreed to accept the research, so the quality of life of the patients was overestimated. Finally, the acceptance period was three months, from 8 March 2021 to 12 May 2021, and was later suspended due to the COVID-19 pandemic. A larger scale of data collection is required to conduct further analyses. We suggest that future research with larger and more diverse samples is necessary to validate and extend our findings.

## 5. Conclusions

Data were collected from lung cancer patients using the QLQ-C30 and the QLQ-LC13 to assess symptom burdens and quality of life. Our findings highlight key areas of symptom burden that affect patients significantly. Future research should focus on developing targeted interventions to address these symptom burdens, particularly appetite loss, fatigue, and dyspnea. Investigating how to effectively manage these symptoms could lead to improved quality of life and potentially enhance survival rates for lung cancer patients. Additional studies should explore the long-term effects of symptom management on overall outcomes in lung cancer patients and assess the effectiveness of various treatment strategies.

## Figures and Tables

**Table 1:** Participant Characteristics (n = 159).

Characteristic		n	%	Global QOL	Cohen’s d
Age, mean ± SD (range)		63.12 ± 11.4 (37–88)			
Sex					
	Male	56	35.2	68.60 ± 21.73	0.05
	Female	103	64.8	67.48 ± 22.61	
Marital status					
	Married	122	76.7	67.14 ± 22.79	0.14
	Single or widowed	37	23.3	70.27 ± 20.46	
Education level					
	College or higher	61	38.4	68.44 ± 21.79	0.04
	High school or lower	98	61.6	67.52 ± 22.62	
Live with family					
	Yes	136	85.5	67.28 ± 22.28	0.18
	No	23	14.5	71.38 ± 22.17	
Duration of postdiagnosis					
	Diagnosed more than five years (before 2016)	50	31.5	67.33 ± 23.86	0.03
	Diagnosed within five years (2017–2021)	109	68.6	68.12 ± 21.57	
Smoking status					
	Never	124	78.0	68.01 ± 23.39	0.03
	Former or current	35	22.0	67.38 ± 17.89	
Family history					
	No	117	73.6	66.52 ± 23.39	0.24
	Yes	42	26.4	71.63 ± 18.41	
Secondhand smoke status					
	No	116	73.0	68.32 ± 22.82	0.08
	Yes	43	27.0	66.67 ± 20.81	
Stage					
	I–III	50	31.5	73.67 ± 17.44	0.41
	IV	109	68.5	65.21 ± 23.73	
Monthly income level					
	≥US$645	95	59.8	69.82 ± 21.06	0.22
	≤US$645	64	40.2	64.97 ± 23.76	
Occupational status					
	Yes	33	20.8	68.69 ± 19.88	0.05
	No	126	79.2	67.66 ± 22.89	
Treatment					
	Nil ^a^	59	37.1	71.05 ± 22.18	0.02 ^b^
	Chemotherapy or radiotherapy	67	42.1	67.29 ± 20.38	
	Chemotherapy and radiotherapy	33	20.8	63.38 ± 25.60	
Treatment line					
	First Line	52	32.7	71.79 ± 20.22	0.04 ^b^
	Second Line	55	34.6	69.85 ± 21.49	
	Third to fifth Line	52	32.7	61.86 ± 24.05	

Abbreviation: QOL, quality of life; ^a^ Nil, no chemotherapy or radiotherapy; ^b^ eta squared.

**Table 2:** Patients’ Quality of Life Score and Lung Cancer Symptom Scales (n = 159).

Characteristic		Mean ^a^	SD
Quality of life (QLQ-C30)			
	Global QOL	67.87	22.24
	Physical function	82.14	20.28
	Role function	82.29	27.98
	Emotional function	86.69	21.26
	Cognitive function	84.28	21.24
	Social function	80.29	27.55
Symptom burden			
QLQ-C30			
	Fatigue	23.27	27.04
	Nausea and vomiting	6.60	18.85
	Pain	16.25	26.65
	Dyspnea	24.53	31.48
	Insomnia	32.08	36.72
	Appetite loss	18.03	30.87
	Constipation	13.42	27.07
	Diarrhea	14.47	26.13
	Financial difficulties	9.64	24.40
QLQ-LC13			
	Dyspnea	19.92	27.28
	Coughing	23.06	29.04
	Haemoptysis	2.10	10.40
	Sore mouth	11.11	25.62
	Dysphagia	5.24	16.58
	Peripheral neuropathy	14.05	24.42
	Alopecia	17.61	31.56
	Pain in chest	10.90	22.66
	Pain in arm or shoulder	10.06	22.74
	Pain in other parts	13.42	27.07

Abbreviations: QOL, quality of life; ^a^ Raw score = *RS* = $\left\{\frac{I1+I2+\ldots +In}{n}\right\};\;Functioning\;scales=1-\left\{\frac{\left(RS-1\right)}{range}\right\}\times 100;$ $Global\;QOL\;and\;symptom\;scales=\{\frac{\left(RS-1\right)}{range}\}\times 100$ ; Quality of life: higher values indicate a higher level of functioning and quality of life; Symptom burden: higher values indicate more perceived health problems.

**Table 3:** The relationship between symptom burden and quality of life (n = 159).

		Global QOL	Physical Function	Role Function	Emotional Function	Cognitive Function	Social Function
QLQ-C30							
	Fatigue	−0.49 **	−0.56 **	−0.66 **	−0.29 **	−0.24 **	−0.52 **
	Nausea and vomiting	−0.29 **	−0.19 *	−0.35 **	−0.19 *	−0.02	−0.21 **
	Pain	−0.36 **	−0.31 **	−0.41 **	−0.17 *	−0.29 **	−0.34 **
	Dyspnea	−0.34 **	−0.48 **	−0.40 **	−0.07	−0.27 **	−0.38 **
	Insomnia	−0.05	−0.11	−0.15	−0.25 **	−0.22 **	−0.12
	Appetite loss	−0.52 **	−0.42 **	−0.52 **	−0.22 **	−0.33 **	−0.46 **
	Constipation	−0.28 **	−0.31 **	−0.29 **	−0.05	−0.17 *	−0.25 **
	Diarrhea	−0.25 **	−0.18 *	−0.22 **	−0.09	−0.16 *	−0.11
QLQ-LC13							
	Dyspnea	−0.44 **	−0.65 **	−0.61 **	−0.15	−0.27 **	−0.51 **
	Coughing	−0.14	−0.23 **	−0.13	−0.11	−0.01	−0.21 **
	Hemoptysis	−0.03	−0.08	0.01	0.04	0.02	−0.09
	Sore mouth	−0.12	−0.19 *	−0.18 *	−0.06	−0.10	−0.22 **
	Dysphagia	−0.01	−0.11	−0.16 *	−0.02	−0.10	−0.20 *
	Peripheral neuropathy	−0.09	−0.06	−0.14	0.08	−0.08	−0.26 **
	Alopecia	−0.17 *	−0.06	−0.05	0.07	−0.09	−0.14
	Pain in chest	−0.37 **	−0.31 **	−0.29 **	−0.22 **	−0.26 **	−0.30 **
	Pain in arm or shoulder	−0.17 *	−0.09	−0.14	−0.02	−0.180 *	−0.16 *
	Pain in other parts	−0.20 *	−0.17 *	−0.31 **	−0.06	−0.27 **	−0.30 **

* *p* < 0.05; ** *p* < 0.01. Abbreviations: QOL, quality of life.

**Table 4:** Results for multivariable regression analyses for quality of life (n = 159).

Quality of Life Model		B	SE	β	t	*p*	Adjusted R^2^	R^2^ Change
Global QOL model								
	Appetite loss	−0.23	0.05	−0.32	−4.38	<0.001	0.27	0.27
	Fatigue	−0.19	0.06	−0.23	−2.95	0.004	0.34	0.07
	Pain in arm or shoulder	−0.15	0.06	−0.15	−2.45	0.016	0.36	0.03
	Dyspnea	−0.15	0.06	−0.18	−2.50	0.013	0.38	0.02
	Alopecia	−0.11	0.04	−0.15	−2.42	0.017	0.40	0.02
Physical function model								
	Dyspnea	−0.33	0.05	−0.45	−6.94	<0.001	0.42	0.42
	Fatigue	−0.23	0.05	−0.31	−4.74	<0.001	0.49	0.07
	Constipation	−0.13	0.04	−0.17	−3.11	0.002	0.51	0.02
	Age	−0.30	0.10	−0.17	−3.05	0.003	0.53	0.03
	Sore mouth	−0.09	0.04	−0.11	−1.98	0.049	0.54	0.01
Role function model								
	Fatigue	−0.36	0.07	−0.35	−5.33	<0.001	0.43	0.43
	Dyspnea	−0.26	0.06	−0.26	−4.05	<0.001	0.52	0.10
	Financial difficulties	−0.27	0.07	−0.24	−3.92	<0.001	0.55	0.03
	Appetite loss	−0.10	0.06	−0.11	−1.67	0.097	0.57	0.02
	Secondhand smoke status yes (versus no)	7.47	3.25	0.12	2.30	0.023	0.58	0.01
	Constipation	−0.14	0.06	−0.14	−2.49	0.014	0.59	0.01
	Smoking status former or current (versus never)	8.08	3.50	0.12	2.31	0.022	0.60	0.01
Emotional function model								
	Fatigue	−0.21	0.06	−0.26	−3.46	0.001	0.08	0.09
	Insomnia	−0.13	0.04	−0.22	−2.84	0.005	0.11	0.04
	RT and CT (versus nil)	8.76	3.93	0.17	2.23	0.027	0.13	0.03
Cognitive function model								
	Appetite loss	−0.11	0.06	−0.15	−1.90	0.059	0.10	0.11
	Financial difficulties	−0.21	0.07	−0.24	−3.06	0.003	0.14	0.05
	Live with family no (versus yes)	−9.91	4.31	−0.16	−2.30	0.023	0.16	0.03
	Income level < 20,000 (versus ≥ 20,000)	8.15	3.18	0.19	2.56	0.011	0.18	0.02
	Insomnia	−0.09	0.04	−0.16	−2.26	0.025	0.20	0.02
	High school or lower (versus college or higher)	−6.50	3.19	−0.15	−2.04	0.043	0.22	0.02
	Dyspnea	−0.11	0.05	−0.16	−2.04	0.043	0.23	0.02
Social function model								
	Fatigue	−0.27	0.08	−0.26	−3.52	0.001	0.27	0.27
	Dyspnea	−0.27	0.07	−0.26	−3.73	<0.001	0.35	0.08
	Peripheral neuropathy	−0.27	0.07	−0.24	−3.99	<0.001	0.39	0.05
	Appetite loss	−0.19	0.06	−0.21	−3.07	0.003	0.42	0.03
	RT and CT (versus nil)	−10.53	4.03	−0.16	−2.61	0.010	0.45	0.02

Abbreviations: QOL, quality of life; RT, Radiotherapy; CT, Chemotherapy; SE, Standard Error.

## Data Availability

The data presented in this article are not readily available because these data are part of an ongoing study, and participants did not consent to their information being publicly shared.
